# Does it make sense to refreeze ovarian tissue after unexpected occurrence of endometriosis when transplanting the tissue?

**DOI:** 10.1186/s13048-022-00972-8

**Published:** 2022-05-05

**Authors:** Anna K. Dietl, Ralf Dittrich, Inge Hoffmann, Dominik Denschlag, Aida Hanjalic-Beck, Andreas Müller, Matthias W. Beckmann, Laura Lotz

**Affiliations:** 1 Department of Obstetrics and Gynecology, Erlangen University Hospital, Friedrich-Alexander University of Erlangen-Nürnberg, Universitätsstrasse 21–23, 91054 Erlangen, Germany; 2 Faculty of Medicine, University of Freiburg, Freiburg, Germany; 3 Center for Gynecologic Endocrinology and Reproductive Medicine, Freiburg, Germany; 4 Department of Obstetrics and Gynecology, Municipal-Hospital, Karlsruhe, Germany

**Keywords:** Fertility preservation, Refreezing, Cryopreservation, Ovarian tissue transplantation, Chemotherapy, Cancer, Iatrogenic ovarian failure, Endometriosis

## Abstract

**Background:**

Ovarian insufficiency is a major concern for long-term cancer survivors. Ovarian tissue cryopreservation for fertility preservation is an emerging technique that has proven successful over the past decade through transplantation of frozen-thawed ovarian tissue. Compared to other established techniques, such as oocyte freezing, ovarian tissue cryopreservation preserves actual organ function and thus the production of sex hormones. Endometriosis in perimenopausal women is rare, however it can be surprising diagnosis in the planned transplantation of cryopreserved ovarian tissue and the already thawed tissue may not be transplanted, so that it has to be refrozen.

**Results:**

Ovarian function returned in the patient two months after transplantation, as shown by estrogen production. Ten months after the ovarian tissue transplantation mild stimulation with FSH was initiated in accordance with a low-dose protocol. When ultrasonography revealed a follicle 17 mm in size in the ovarian graft, hCG was added and after follicular puncture one oocyte was obtained. The oocyte could be fertilized by IVF and transferred to the uterus. On day 14 after embryo-transfer, a positive hCG-Level was detected and after an uncomplicated pregnancy a healthy child was delivered.

**Conclusions:**

We report the first pregnancy and live birth achieved using transplantation of thawed and refrozen ovarian tissue in a woman treated by chemotherapy and subsequent endometriosis surgery. Refreezing of cryopreserved ovarian tissue is not a hindrance to successful transplantation of ovarian tissue. Against the background of increasing numbers of candidates for transplantation of ovarian tissue is expected that the combination chemotherapy followed by endometriosis will increase.

## Background

Since the first report on a live birth after thawing and transplantation of ovarian tissue [1], this method has spread increasingly worldwide. Meanwhile, a success rate of about 30% can be achieved after autotransplantation of cryopreserved ovarian tissue [2, 3]. Although the survival rate in cancer patients has been significantly improved by chemotherapy, ovarian toxicity remains a major problem. Gonadotoxic chemotherapy during reproductive age can induce a primary ovarian insufficiency and reduction of follicular reserve.

Ovarian tissue cryopreservation has several advantages over embryonic or oocyte cryopreservation and is the only fertility maintenance option for children, adolescents, and young adult cancer patients who require immediate chemotherapy and do not have enough time for ovulation induction. The procedure is independent of a menstrual cycle. A large number of oocytes and primordial follicles can be preserved. The hormonal function of the ovary can be restored and this technique does not require ovarian stimulation or a sperm donor [4].

Besides breast cancer, Hodgkin’s lymphoma is the most common indication for ovarian tissue transplantation. Since toxic chemotherapy reduces the ovarian reserve and different potential protectants do not exist, ovarian tissue cryopreservation remains the method of choice besides embryo and oocyte freezing [5].

A benign disease that can also reduce fertility is endometriosis. The success rates of assisted reproductive techniques remains low [6]. It consists of the surgical risk for altering ovarian tissue and premature ovarian failure [7]. Endometriosis in perimenopausal women is rare and chemotherapy can induce perimenopausal status in women of reproductive age [8].

Herein, we report a live birth after ovarian tissue transplantation in a survivor of Hodgkin lymphoma, who developed surprisingly endometriosis fourteen years later. This is the first live birth after thawed and refreezed ovarian tissue transplantation in which the patient was treated by chemotherapy and later on by endometriosis surgery.

## Results

The preoperative hormonal levels were in the perimenopausal range. Two months after transplantation, a fall in gonadotropin and a rise in E_2_ were detected. The patient reported regular but shortened menstrual cycles of 22 – 24 days. At the patient’s request, cycle monitoring was performed. During the fifth menstrual cycle, ovulation was triggered with hCG for IVF. But spontaneous ovulation occurred immediately before follicular puncture.

Ten months after the ovarian tissue transplantation follicular maturation was stimulated by 100 IU FSH (Gonal F) and GnRH-antagonist (Ganirelix). When ultrasonography revealed a follicle 17 mm in the ovarian autograft, hCG was added and after follicular puncture one oocyte could be obtained. The oocyte could be fertilized by IVF and transferred to the uterus on day 3 (8-cell-stage). Progesterone was administered to support the luteal phase. On day 14 after embryo transfer, a positive hCG-level was revealed and a clinical pregnancy was later confirmed on vaginal ultrasonography. The patient delivered a healthy female child by cesarean section after uncomplicated pregnancy (2860 g, 38 weeks of gestation).

## Discussion

Over the last few years, there has been a significant increase in the number of patients using reproductive techniques, especially in young cancer patients. Fertility preserving is also possible with ovarian tissue cryopreservation, which can no longer be considered an experimental procedure [9]. Despite these promising results and the increasing interest in this method, cryopreservation and transplantation is a complex procedure that requires experience and validation of the techniques.

The present report describes the first live birth after transplantation of thawed and refrozen ovarian tissue following chemotherapy and consecutive endometriosis surgery. The ovarian tissue was frozen for 14 years and during this time a pelvic endometriosis developed. Only after surgical repair of the endometriosis the ovarian tissue was transplanted and pregnancy at the 2^nd^ IVF attempt could be achieved, resulting in a live-birth. Because of the extensive pelvic endometriosis the ovarian tissue had to be frozen twice.

Since Donnez et al. [1] described the first live birth in 2004 after orthotopic transplantation of cryopreserved ovarian tissue, a central point of discussion remains the endogenous reactivation of the ovaries by the retransplanted ovarian tissue and whether the follicles leading to pregnancy originate from the grafts or the ovaries. In our patient's case, the pregnancy-originating follicle was obtained directly by follicular puncture of the ovarian graft. The ovaries did not show any follicular development.

So far, one report by Kristensen et al. describes the refreezing of already transplanted tissue in the xenomodel [10]. This patient – treated for early-stage ovarian cancer – underwent ovarian tissue cryopreservation and subsequent heterotopic transplantation 9 years after freezing. After a successful IVF twin pregnancy, grafted tissue was removed for safety reasons and refrozen. The histologic evaluation of the xenograft revealed surviving pre-antral stage follicles. This xenografting and the present report are important proofs of concept that human ovarian tissue can be frozen twice.

Endometriosis remains difficult to diagnose, because of no biomarkers are available. It is estimated to effect 10% of reproductive-age women and the prevalence ranges from 5 to 50% among infertile women [11]. The postulated origins of endometriosis are retrograde menstruation, coelomic metaplasia, and lymphatic and vascular metastasis. In studies of repeat surgeries, endometriosis lesions progressed (29%), regressed (42%), or were stable (29%), according to r-ASRM staging [12].

The fact of endometriosis in perimenopausal women is very rare: the patient in the present case showed perimenopausal hormonal levels at the time of planned retransplantation. During reproductive age it is well known that endometriosis shows an estrogen-dominated syndrome [13, 14]. The underlying mechanism of endometriosis in the peri- or postmenopausal period is not known. Estrogen-production during menopause could be caused by extraovarian organs: adrenal gland, endometrial stroma, adipose tissue and skin [15]. Another estrogen-production could be caused by hormonal replacement therapy, which can induce endometriosis and create new implants [14]. Our patient did not take any hormonal replacement therapy except a combined oral contraceptive pill until 2014. No endometriosis was visualized laparoscopically when ovary tissue was harvested in 2004.

Up to now, three reports on ovarian cryopreserved transplantation in endometriosis have been published [16]: Donnez et al. [17] reported for the first time on two patients with endometriosis without ovaries damaged by cancer chemotherapy. A unilateral oophorectomy and orthotopic reimplantation of fresh ovarian tissue resulted in pregnancy after 3^rd^ IVF attempt in one case and normal ovarian tissue with endocrine reactivation in the second one. Oktay and Oktem [18] reported a patient with endometriosis and no history of cancer-chemotherapy. She underwent a unilateral oophorectomy and a cryopreservation of the ovarian cortex and six months later an orthotopic transplantation. Three months later, the graft showed normal ovarian tissue with recovery of ovulation. In another case the orthotopic reimplantation failed and a second, heterotopic, transplantation was performed on the abdominal wall, but 12 months after the second transplantation no ovarian function recovery could be detected [19].

Chemotherapy and endometriosis compromise ovarian reserve and fertility by several mechanisms [5, 20]. The mean age of patients undergoing ovarian tissue cryopreservation is about 22 years [21], at diagnosis of endometriosis about 28 years [22]. The occurrence of endometriosis is therefore possible in the interval between ovarian tissue harvesting, oncological therapy and transplantation. The challenge for the clinician is to perform the transplantation at the most optimal time and, if possible, to reduce the appearance of endometriosis in the abdominal cavity. Extensive endometriosis can be a surprising diagnosis during the intended laparoscopic transplantation of cryopreserved ovarian tissue. The thawed tissue may then not be transplanted and must be refrozen until the next attempt after the endometriosis has been treated. Ovarian tissue obviously has no damage from this twice freezing, but the follicular density was not histologically evaluated. Follicular survival rate after a single freezing – thawing procedure is 70–80% [23]. This tolerance of human ovarian tissue is apparently sufficient to make a successful second attempt of a freezing – thawing procedure.

Since the technique of ovarian tissue transplantation is becoming increasingly accepted, the combination of ovarian tissue cryopreservation – oncological therapy – transplantation of ovarian tissue is likely to become more important. In order to achieve an optimal treatment, the implementation of oncofertility services to women is an essential step. To provide fertility preservation strategies to prepubertal and young women with cancer and/or endometriosis, each medical institution should have a highly experienced oncofertility team which consists of medical oncologists, gynecologists, reproductive biologists, and research scientists.

## Conclusion

In conclusion, it has been shown that after sequential therapy (chemotherapy of Hodgkin-lymphoma, endometriosis surgery) the transplantation of thawed and refreezed cryopreserved ovarian tissue can achieve pregnancy. Given the increasing number of pregnancies and births after ovarian transplantation, it is expected that the combination of chemotherapy/endometriosis will also increase. Although the road is mapped out, further studies are needed to make a general recommendation.

## Material and methods

### Patient information

A 19-year-old woman was diagnosed with nodular sclerosing Hodgkin lymphoma, clinical stage IIIA. The patient received the chemotherapy regimen BEACOPP-14 (bleomycin, etoposide, adriamycin, cyclophosphamide, vincristine, procarbazine, prednisone) developed by the German Hodgkin Study Group for patients with unfavorable risk factors.

Before the start of this treatment, fragments of the ovarian cortex were removed laparoscopically from both ovaries. The systematic laparoscopic view of bilateral ovaries, fallopian tubes, uterus, peritoneal surfaces, and diaphragm surfaces was unaffected.

Fourteen years after complete remission the 34-year-old patient request ovarian tissue transplantation due to an unfulfilled desire to have children. After chemotherapy she took oral contraception pill, but no hormonal replacement therapy. After discontinuing the contraception pill, the patient had an irregular menstrual cycle between 22 and 28 days. Hormonal laboratory results showed perimenopausal levels (FSH: 13–47 mlU/ml, LH: 7–16 mlU/l, AMH: 0,05 µg/l).

The patient did not complain about typical endometriosis symptoms like dysmenorrhea, dysuria, dyschezia and dyspareunia.

### Ovarian tissue freezing

Ovarian tissue was frozen directly after laparoscopic harvest using a slow freezing protocol that used the combination of 1,2-propanediol and sucrose as a cryoprotectant. The detailed protocol for freezing and thawing of the ovarian cortex has been described previously [24].

### Ovarian tissue refreezing and endometriosis surgery

**Fig. 1:**
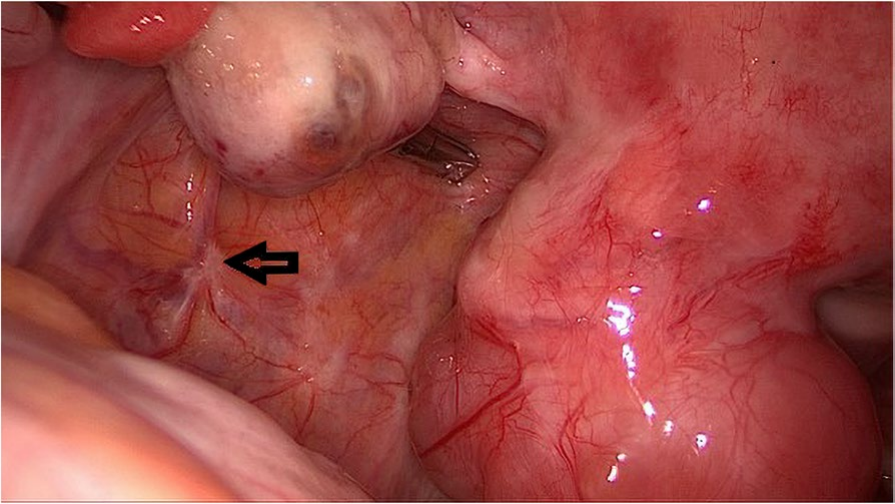
Retroovarian endometriotic lesion with scarring in the fossa ovarica (arrow)

The patient underwent a laparoscopy with the intention of transplanting the ovarian tissue. Therefore the tissue was thawed according the protocol [25, 26]. However, extensive endometriosis at pelvic walls (especially at both fossae ovaricae, Fig. 1), pouch of Douglas, and bladder peritoneum was diagnosed (r-ASRM stage II). Because of these lesions the original retransplantation-surgery was canceled and the ovarian tissue was refrozen.

Thawed ovarian tissue was refrozen using a slow freezing protocol with ethylene glycol and sucrose as a cryoprotectant [27, 28].

In a laparoscopical reoperation the endometriosis-lesions were completely removed by predominantly retroperitoneal dissection. The histopathological examination confirmed the endometriotic lesions.

### Autologe ovarian tissue transplantation

Four months after endometriosis surgery, the patient requested the autotransplantation of the twice-cryopreserved ovarian tissue. The thawed tissue was transplanted into a peritoneal pocket of the right ovarian fossa. Both tubes were patent in the chromopertubation.

The detailed patient history is outlined in Table 1.

**Table 1:** Outline of patient history

Year	Patient history
2004	Feb: Diagnosed with stage III A Hodgkin lymphoma March: Ovarian tissue cryopreservation Chemotherapy regimen BEACOPP-14 July: Patient was perimenopausal
2018	Nov: Ovarian tissue thawed and prepared for transplantation Laparoscopy: extensive endometriosis (patient not informed about endometriosis surgery) Ovarian tissue refreezed Dec: Laparoscopy: endometriosis-lesions were completely removed
2019	May: Autotransplantation of the refreezed ovarian tissue Oct: Started IVF-treatment
2020	March: Pregnancy after IVF
	Nov: Birth of a healthy girl

## Data Availability

Full availability of data and material are available from the corresponding author on reasonable request.

## Abbreviations

IVF: In vitro fertilisation
hCG: Human-chorionic gonadotropin
FSH: Follicle stimulations hormone
LH: Luteinizing hormone
mIU: Milli-international Unit
IU: International Unit
AMH: Anti-Müllerian-hormone
